# EasyGDB: a low-maintenance and highly customizable system to develop genomics portals

**DOI:** 10.1093/bioinformatics/btac412

**Published:** 2022-06-24

**Authors:** Noe Fernandez-Pozo, Aureliano Bombarely

**Affiliations:** Departamento de Fruticultura Subtropical y Mediterránea, Instituto de Hortofruticultura Subtropical y Mediterránea La Mayora (IHSM-CSIC-UMA), Málaga 29010, Spain; Department of Bioscience, Universita degli Studi di Milano, Milan 20133, Italy; Department of Bioscience, Universita degli Studi di Milano, Milan 20133, Italy; Departamento de Biotecnología y Mejora Vegetal de Especies Cultivadas, Biología Molecular y Celular de Plantas (IBMCP), UPV-CSIC, Valencia 46022, Spain

## Abstract

**Summary:**

EasyGDB is an easy-to-implement low-maintenance tool developed to create genomic data management web platforms. It can be used for any species, group of species, or multiple genome or annotation versions. EasyGDB provides a framework to develop a web portal that includes the general information about species, projects and members, and bioinformatics tools such as file downloads, BLAST, genome browser, annotation search, gene expression visualization, annotation and sequence download, and gene ids and orthologs lookup. The code of EasyGDB facilitates data maintenance and update for non-experienced bioinformaticians, using BLAST databases to store and retrieve sequence data in gene annotation pages and bioinformatics tools, and JSON files to customize metadata. EasyGDB is a highly customizable tool. Any section and tool can be enabled or disabled like a switch through a single configuration file. This tool aims to simplify the development of genomics portals in non-model species, providing a modern web style with embedded interactive bioinformatics tools to cover all the common needs derived from genomics projects.

**Availability and implementation:**

The code and manual to use EasyGDB can be found at https://github.com/noefp/easy_gdb.

## 1. Introduction

The advances in sequencing technologies and assembly tools have highly reduced the cost and difficulty of genome sequencing and assembly in the last two decades. It allowed an exponential availability of genome sequences and the emergence of projects to sequence thousands of genomes (Cheng *et al.*, 2018; Grigoriev *et al.*, 2014; Rhie *et al.*, 2021). However, in non-model species, new genomes and annotations are not always available after publication, consequently, only raw data, or data with no annotations can be found (Strijk *et al.*, 2021; Zhang *et al.*, 2020). In other cases, public databases such as GenBank do not meet some of the needs of the community behind these genomes. Often, web portals and genomic databases and tools are an important part of the development of genome sequencing projects in which the community plays an essential part in the curation of the data.

The available systems to implement genomics portals are usually complex and hard to maintain in the long term. In some cases, the installation process is complicated and requires many dependencies. In others, very complex database schemas are hard to maintain and to populate, requiring to fill tables that sometimes might not be needed for all projects. One of the challenges of genomics databases is their maintenance because of the difficulty to upload new data and to keep them up to date. Often some genomics databases are not updated for years or have to stop their service after some time (Delmans *et al.*, 2017; Fernandez-Pozo *et al.*, 2022). For these reasons, we present EasyGDB, an easy-to-implement, maintain and customize system to develop genomics databases, which includes a set of useful bioinformatics tools to analyze, visualize and search the data.

## 2. Materials and methods

EasyGDB can be easily installed using the Docker-compose installation file available in Github (https://github.com/noefp/easyGDB_docker). This provides containers with pre-installed Apache server, PostgreSQL and PHP. The annotation database stores gene names, versions, species and annotations in a simple PostgreSQL schema. The application code is written in PHP and the front-end uses CSS, JavaScript, JQuery, DataTables, Bootstrap 4, Apexcharts and HTML5 canvas. Sequences are stored as BLAST databases, allowing sequence similarity searches, sequence retrieval and sequence visualization. JSON files are used to control metadata customization. EasyGDB code was developed by incremental improvement of the code from other sites such as the PpGML DB (Fernandez-Pozo *et al.*, 2020), OliveTreeDB (Jiménez‐Ruiz *et al.*, 2020) and the Aethionema *arabicum* DB (Fernandez-Pozo *et al.*, 2021), and BLAST output interface is based on the Sol Genomics Network code (Fernandez-Pozo *et al.*, 2015).

## 3. Results

EasyGDB provides a highly customizable and low-maintenance system to easily develop genomics web portals with bioinformatics tools.

### 3.1 EasyGDB bioinformatics tools and other features

EasyGDB can host genomics projects with single or multiple species or annotation versions. It contains tools such as (i) BLAST, (ii) keyword search by annotations and gene IDs, (iii) genome browser (JBrowse), (iv) gene list annotation download, (v) gene list sequence download, (vi) gene expression visualization tools and (vii) gene version and orthologs lookup tool. Genes in JBrowse, BLAST and search results, are linked to dynamic gene annotation pages, which display a frame with the genome browser, and the available annotations and sequences. Additionally, tools such as Apollo (Dunn *et al.*, 2019) can be implemented for gene manual curation. Examples of EasyGDB features and comparison with other tools are available in Supplementary File S1.

### 3.2 Easy customization

**Fig. 1.**
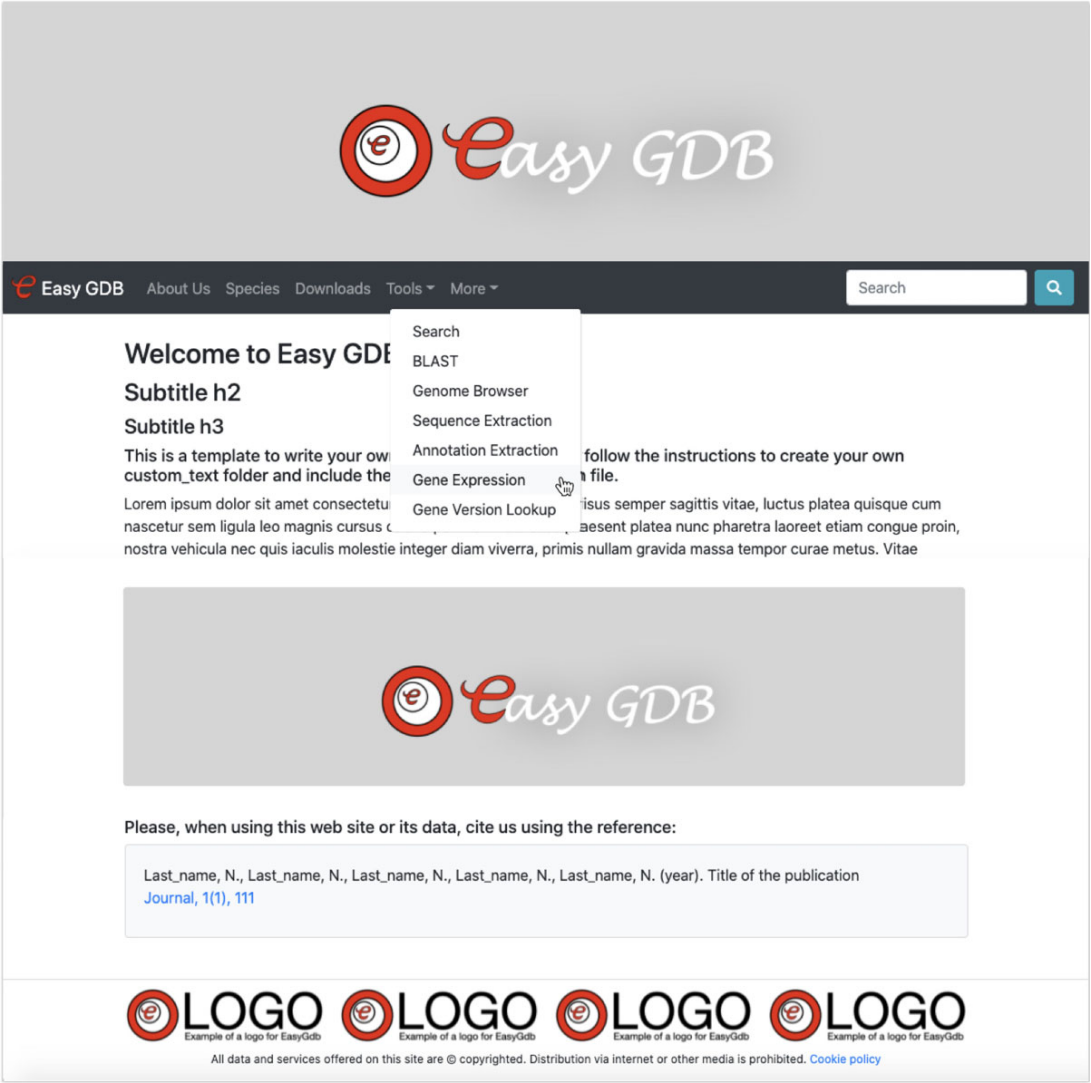
EasyGDB template before customization. Images, logos, site name and home page text are easily customizable. Elements in the menu toolbar can be enabled or disabled

EasyGDB includes customizable templates for common sections, such as ‘home page’, ‘about us’, ‘downloads’, ‘species’, ‘tools’ and custom pages, which can be enabled or disabled like a switch in a single configuration file (Fig. 1). The application name, images, logos and text in the PHP template can be replaced by the desired ones. Simple JSON files facilitate the information for project metadata. For example, one file controls the links to the annotation sources, allowing a great flexibility to upload annotation of any type, which will be linked to their source repository in the gene annotations page (e.g. UniProt, InterProScan, NCBI, etc.).

### 3.3 Low maintenance

To simplify maintenance, sequences are stored in BLAST databases. Then, just placing the BLAST DB files in the *blast_dbs* directory will automatically display the available sequence sets in the BLAST tool menu, the sequence extraction tool and the gene annotation page. Moreover, managing file downloads is very simple, just placing a file in the download folder will automatically show it on the web. EasyGDB code replicates the downloads directory structure in the downloads page, allowing any folder and subfolder organization, e.g. by species, by data type, by project, etc. Additionally, annotations are stored in a simple database schema, which facilitates data management.

### 3.4 Easy implementation

Docker-compose files are available for an easy installation of the dependencies needed (https://github.com/noefp/easyGDB_docker), and implementation instructions, with and without Docker, can be found in GitHub together with the code (https://github.com/noefp/easy_gdb). Perl scripts are provided to import annotation data in the database and to import tracks in JBrowse.

Using Docker, it is possible to install EasyGDB in a personal computer, which can be useful to manage annotation data from multiple projects or versions, controlled in a single implementation with several configuration files.

More information about EasyGDB installation, customization and about its tools and features, can be found in its manual (https://github.com/noefp/easy_gdb#readme) and in this playlist of video tutorials (https://youtube.com/playlist?list=PL7jt0JZOquU7nAkIfbJN2jmnExeKC6Qpq). An example of a web portal implemented using easyGDB can be found at https://mangobase.org.

## Funding

This work was supported by the Junta de Andalucía Emergia program [EMERGIA20_00286], Ministerio de Ciencia e Innovación [RYC2020-030219-I] and USDA NIFA [2020-51181-32198].

*Conflict of Interest*: none declared.

## Supplementary Material

btac412_Supplementary_Data
