# Supplementary material for: EasyGDB: a low-maintenance and highly customizable system to develop genomics portals
Source: Bioinformatics. 2022 Jun 24;38(16):4048–50. doi: 10.1093/bioinformatics/btac412 (PMC9364376; doi:10.1093/bioinformatics/btac412)
Supplement: btac412_Supplementary_Data [file btac412_supplementary_data.pdf]

Supplemental Material – EasyGDB, a low-maintenance and highly customizable system to develop genomics portals.

Features of EasyGDB in comparison with other tools

The philosophy of EasyGDB is to provide a simple system to create genomics portals with a set of bioinformatics tools to get all the information needed from the data while keeping the installation and maintenance process very simple and affordable for most users with basic knowledge of bioinformatics. EasyGDB could be useful for a wide range of researchers, from predoctoral students with basic knowledge of bioinformatics, who could install it locally in their laptop and easily maintain the data they are working with, to more advanced users with basic knowledge of bioinformatics who could implement it in a server. There are other tools to develop genomics portals, but they are not focused on simplifying maintenance. For example, Tripal (http://tripal.info/) (Spoor *et al.*, 2019) is a widely used and very complete system, which provides many modules and a large database schema to cover genomics tools for many tasks, but it is more complex and harder to maintain. This system requires skilled administrators and more time to be implemented and maintained. However, in many cases, genomics portals, especially for non-model species, do not need or contain data to use many of its features. Another interesting tool is CoGe (https://genomevolution.org/coge/) (Lyons and Freeling, 2008), which provides a web service to upload, visualize and share genomics data. Also interesting are tools such as Apollo (http://genomearchitect.github.io/) (Dunn *et al.*, 2019), yrGATE (http://www.plantgdb.org/prj/yrGATE/) (Wilkerson *et al.*, 2006) and ORCAE (https://bioinformatics.psb.ugent.be/orcae/) (Sterck *et al.*, 2012), which are mainly designed for visualization and manual curation of gene annotations.
EasyGDB is compatible with GMOD tools such as Apollo and JBrowse. In easyGDB it is possible to enable Jbrowse as the default genome browser, and in case gene annotation manual curation is desired, Apollo is perfectly compatible and would be the preferable tool for this task. On the other hand, tools such as genSAS (https://www.gensas.org/) (Humann *et al.*, 2019), InterProScan (Jones *et al.*, 2014) and Diamond (Buchfink *et al.*, 2015), provide gene annotation tools and pipelines, and could be used to annotate the data that will be included in EasyGDB.

EasyGDB provides bioinformatics tools such as the expression atlas, the gene ID lookup, and the gene list annotation and sequence download, which are not available in most of the previously mentioned software (Table S1). Only Tripal has modules to implement somehow similar tools to fulfill the features available in EasyGDB, but in most cases, they are harder to implement and maintain, and might require installing more dependencies and to populate a large relational database. Essential bioinformatics tools such as BLAST, genome browser, file download and dynamic gene annotation pages, are commonly available in the compared software (Table S1). On the other hand, as the compared software in some cases have different purposes, they might have many other features not available in EasyGDB.

Table S1. Features of EasyGDB in comparison with other tools

	EasyGDB	Tripal	ORCAE	Apollo	yrGATE	CoGe	genSAS
BLAST/BLAT	Yes	Yes	Yes	Yes	Yes	Yes	Yes
Genome browser	Yes	Yes	Yes	Yes	Yes	Yes	Yes
Gene manual curation	Yes*1	Yes	Yes	Yes	Yes	No	Yes*1
Expression Atlas	Yes	Yes	No	No	No	No	No
Annotation/gene ID Search	Yes	Yes	Yes	ID	No	ID	ID*1
File Download	Yes	Yes	Yes	Yes	Yes	Yes	Yes
Gene selection annotation download	Yes	Yes	No	No	No	No	No
Gene selection sequence download	Yes	Yes	No	No	No	No	No
Gene annotation page	Yes	Yes	Yes	Yes	Yes	Yes	Yes*1
Gene selection ID lookup	Yes	Yes	No	No	No	No	No
Customizable style	Yes	Yes	No?	No	No	No	No
Customizable Web templates	Yes	Yes	No	No	No	No	No
Docker installation	Yes	Yes	No	Yes	No	No	No

*1It is possible using Apollo and similar tools. Green, orange and red colors highlight presence, partial and absence of features respectively.

Examples of available features in EasyGDB

The figures S1 to S13 below, show examples of the available tools in EasyGDB.

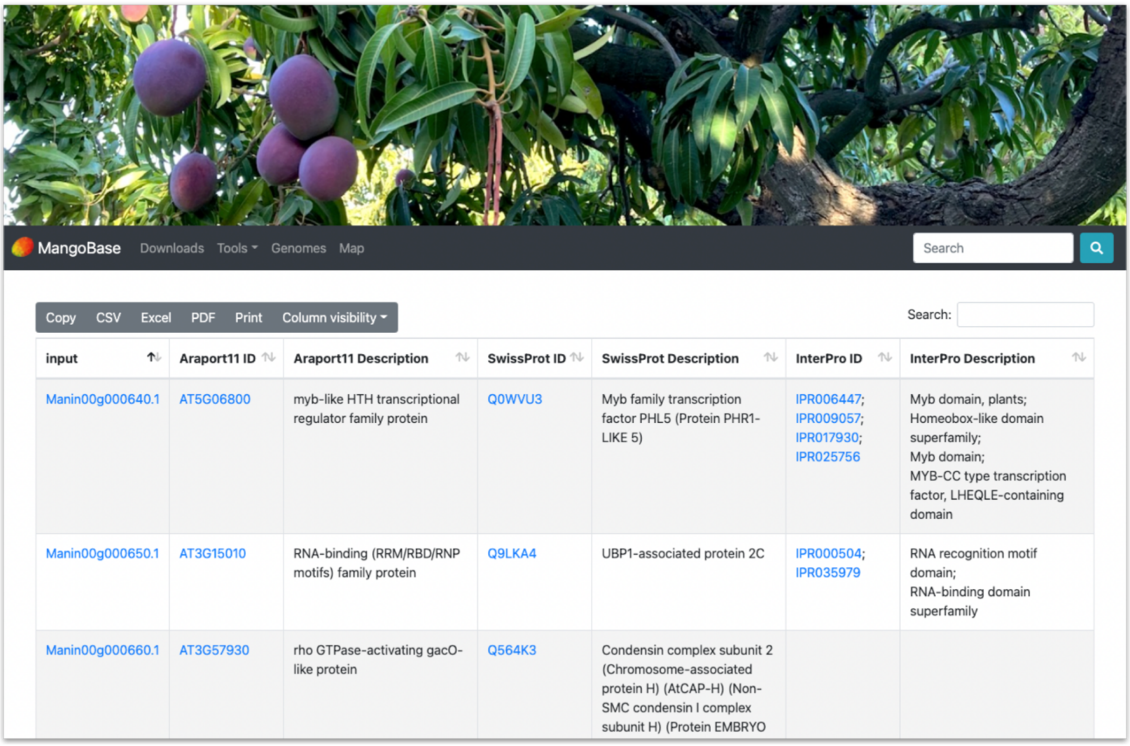

Figure S1. Example of the Annotation Extraction Tool in MangoBase. All annotations for a list of genes are shown in a downloadable table. A configuration JSON file can be used to define external links to annotation databases.

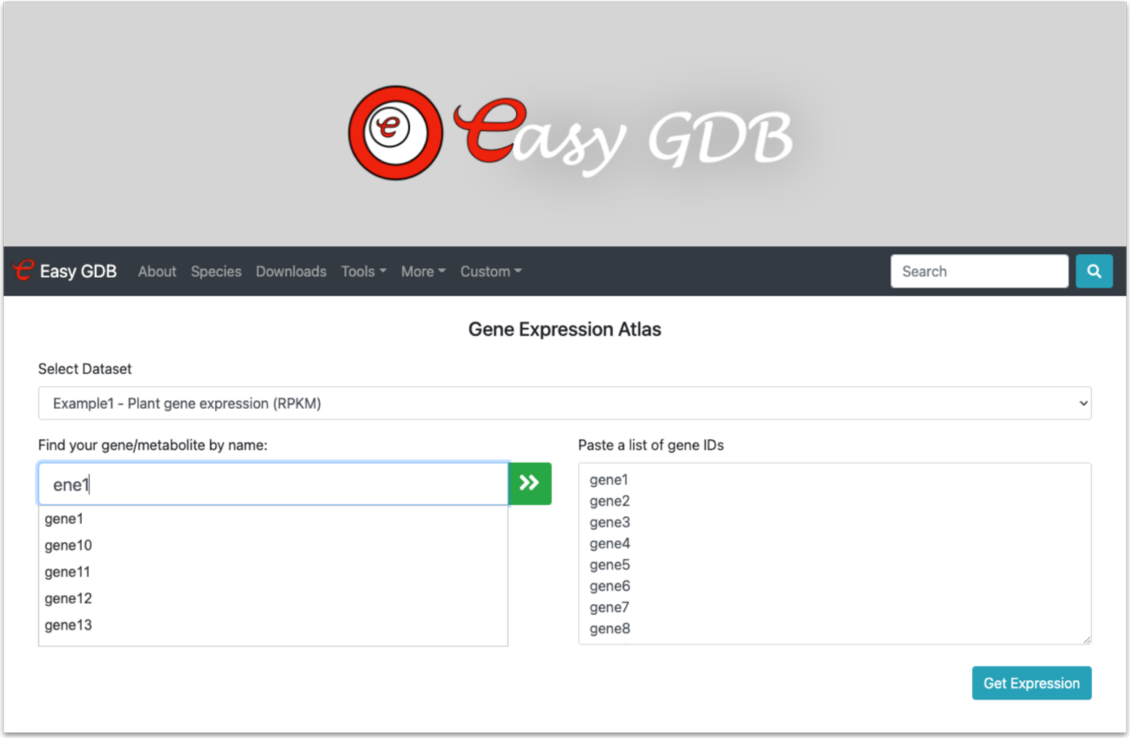

Figure S2. EasyGDB Expression Atlas input page. A list of genes can be directly pasted on the right panel or genes can be written one by one with the help of the autocomplete assistant, which facilitates finding gene, protein and metabolite names.

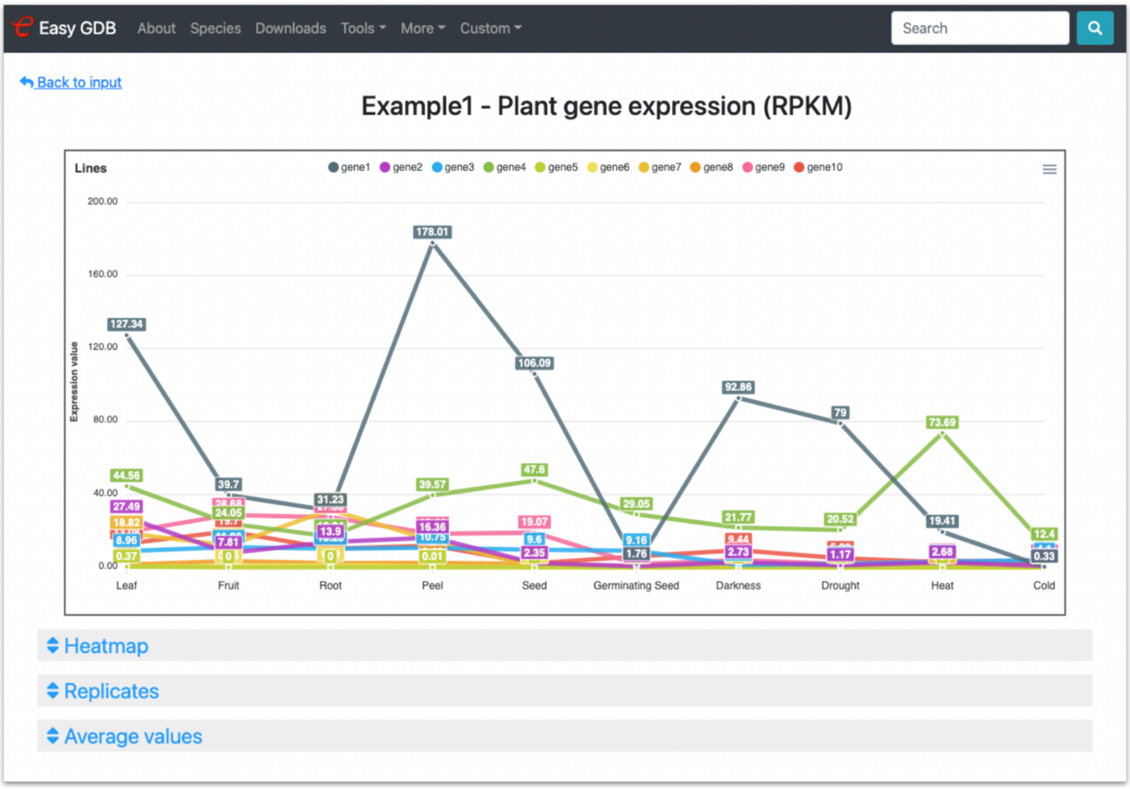

Figure S3. EasyGDB interactive expression atlas line plot. Selected genes can be hidden/shown and highlighted for a better visualization.

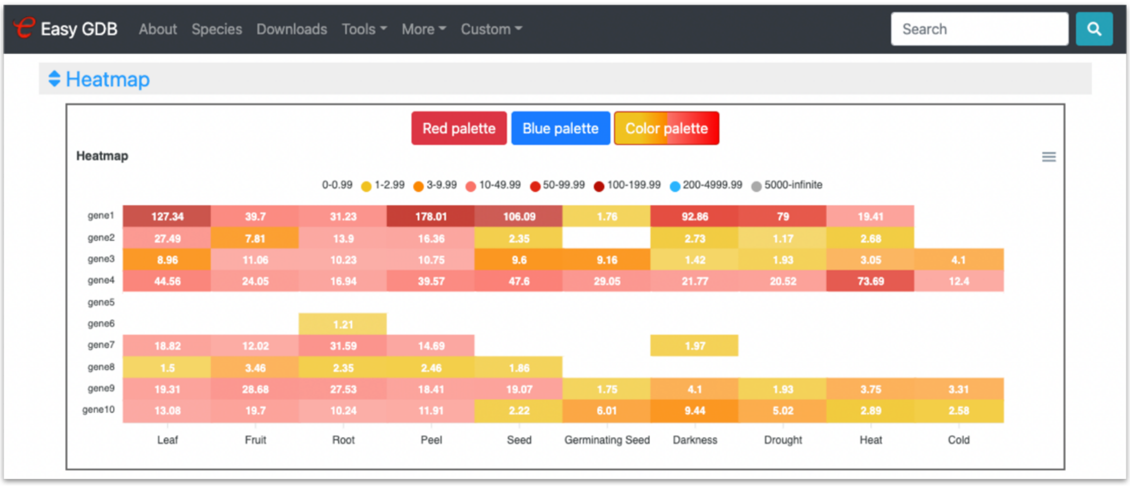

Figure S4. EasyGDB interactive expression atlas heatmap. Multiple color scales can be chosen and it is possible to interact with the color scale ranges to display only boxes within the selected range.

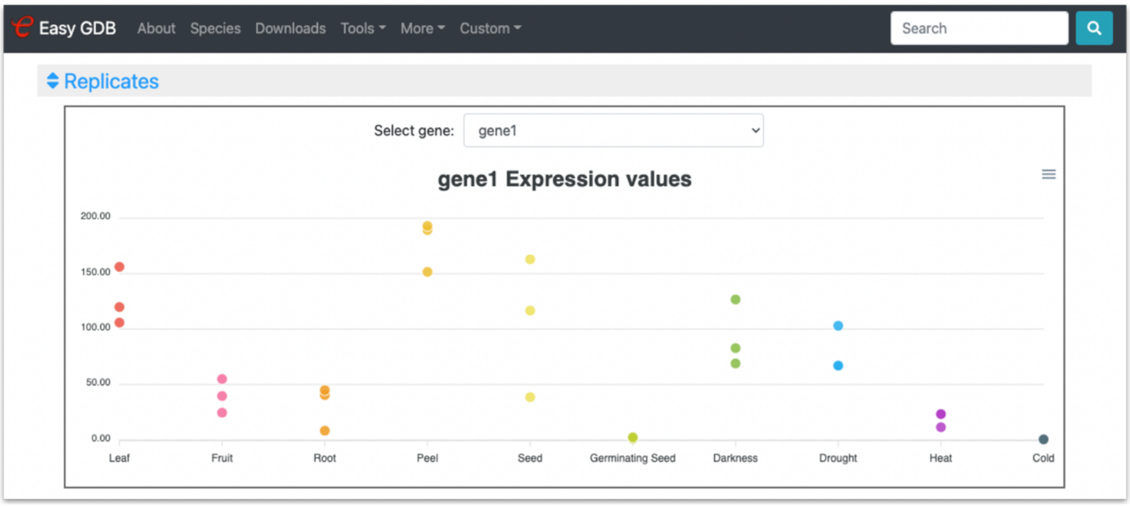

Figure S5. EasyGDB interactive expression atlas replicates plot. Expression values for all replicates are shown for all samples of the selected gene.

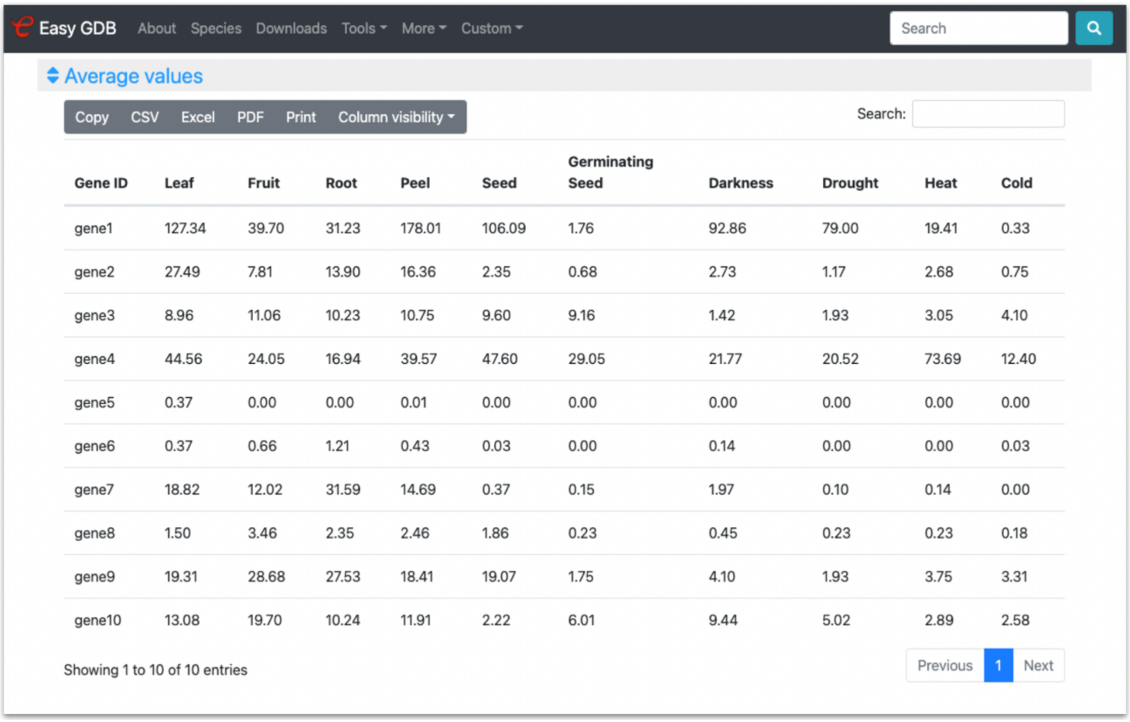

Gene ID	Leaf	Fruit	Root	Peel	Seed	Germinating Seed	Darkness	Drought	Heat	Cold
gene1	127.34	39.70	31.23	178.01	106.09	1.76	92.86	79.00	19.41	0.33
gene2	27.49	7.81	13.90	16.36	2.35	0.68	2.73	1.17	2.68	0.75
gene3	8.96	11.06	10.23	10.75	9.60	9.16	1.42	1.93	3.05	4.10
gene4	44.56	24.05	16.94	39.57	47.60	29.05	21.77	20.52	73.69	12.40
gene5	0.37	0.00	0.00	0.01	0.00	0.00	0.00	0.00	0.00	0.00
gene6	0.37	0.66	1.21	0.43	0.03	0.00	0.14	0.00	0.00	0.03
gene7	18.82	12.02	31.59	14.69	0.37	0.15	1.97	0.10	0.14	0.00
gene8	1.50	3.46	2.35	2.46	1.86	0.23	0.45	0.23	0.23	0.18
gene9	19.31	28.68	27.53	18.41	19.07	1.75	4.10	1.93	3.75	3.31
gene10	13.08	19.70	10.24	11.91	2.22	6.01	9.44	5.02	2.89	2.58

Figure S6. EasyGDB expression atlas average values table.

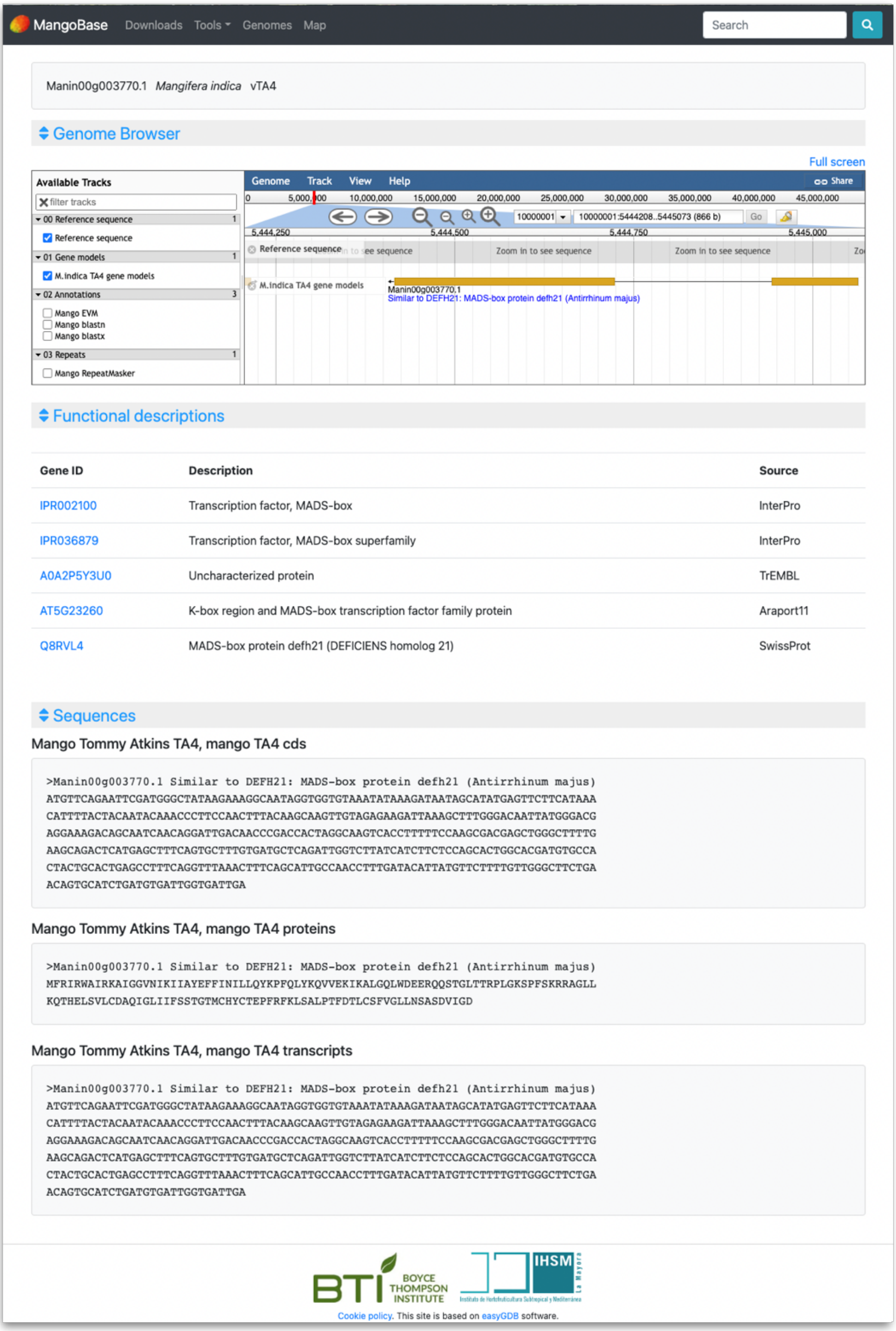

Figure S7. Example of a gene annotation page in MangoBase, implemented with EasyGDB. A configuration JSON file can be used to define external links to annotation databases. the displayed gene sequences are automatically extracted from the BLAST databases.

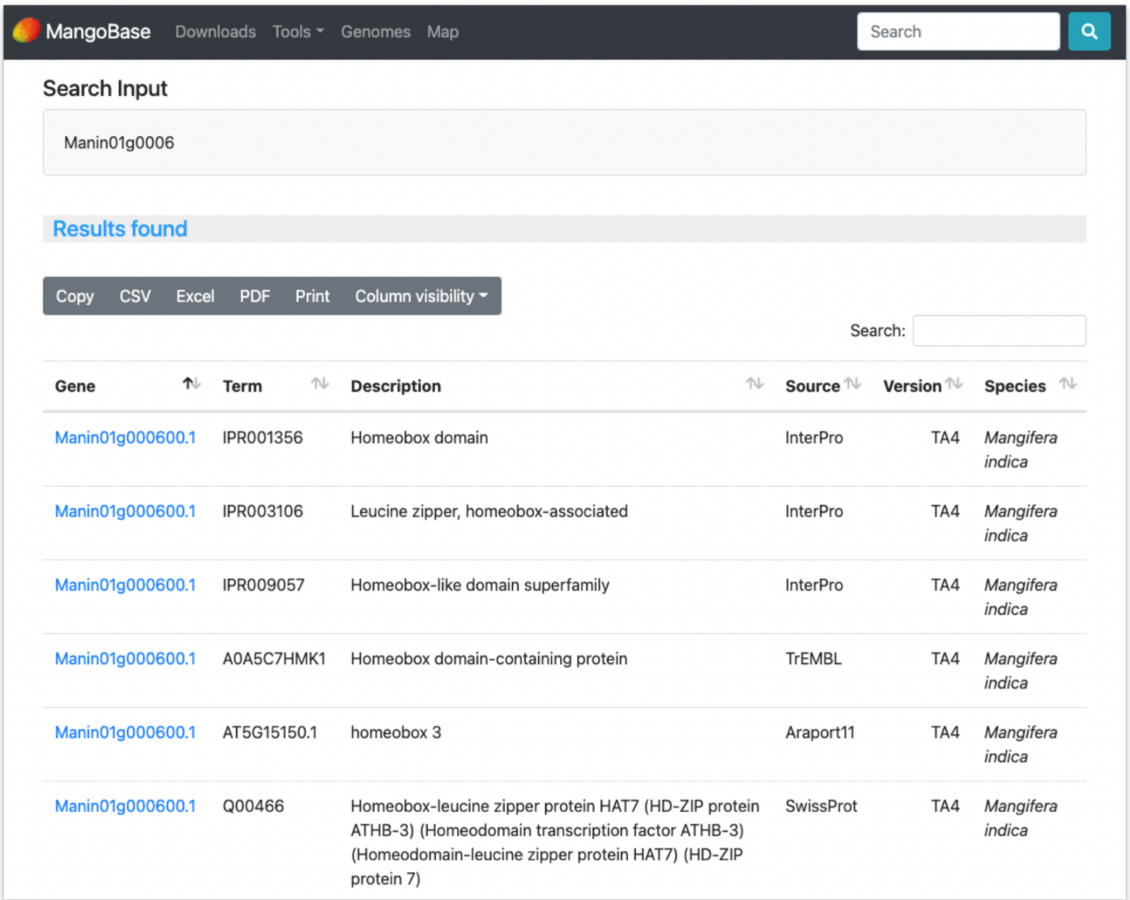

Gene	Term	Description	Source	Version	Species
Manin01g000600.1	IPR001356	Homeobox domain	InterPro	TA4	*Mangifera indica*
Manin01g000600.1	IPR003106	Leucine zipper, homeobox-associated	InterPro	TA4	*Mangifera indica*
Manin01g000600.1	IPR009057	Homeobox-like domain superfamily	InterPro	TA4	*Mangifera indica*
Manin01g000600.1	A0A5C7HMK1	Homeobox domain-containing protein	TrEMBL	TA4	*Mangifera indica*
Manin01g000600.1	AT5G15150.1	homeobox 3	Araport11	TA4	*Mangifera indica*
Manin01g000600.1	Q00466	Homeobox-leucine zipper protein HAT7 (HD-ZIP protein ATHB-3) (Homeodomain transcription factor ATHB-3) (Homeodomain-leucine zipper protein HAT7) (HD-ZIP protein 7)	SwissProt	TA4	*Mangifera indica*

Figure S8. Example of the search result in MangoBase, which is implemented with EasyGDB.

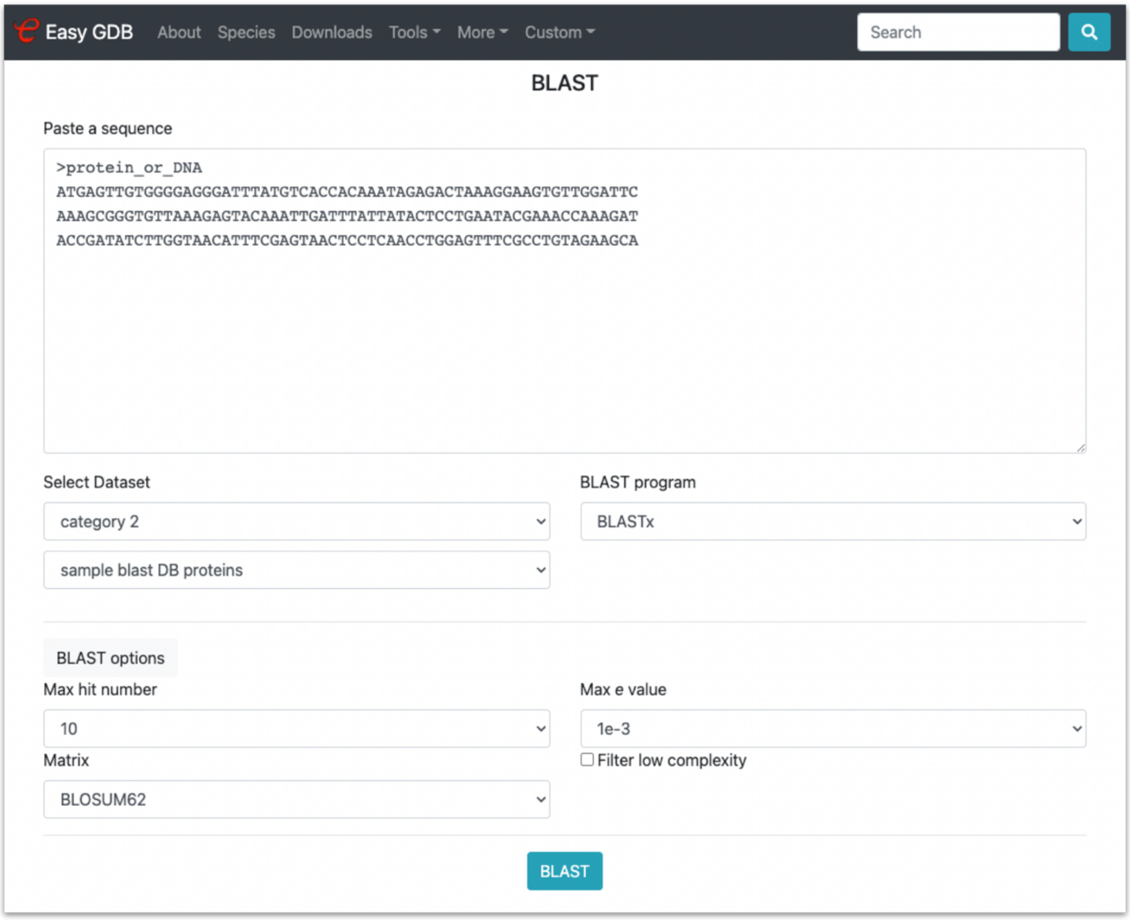

Figure S9. EasyGDB BLAST input page. Available databases appear and disappear automatically just by placing them in the blast_db path. Blast databases are organized in folders that define categories in the input select menu.

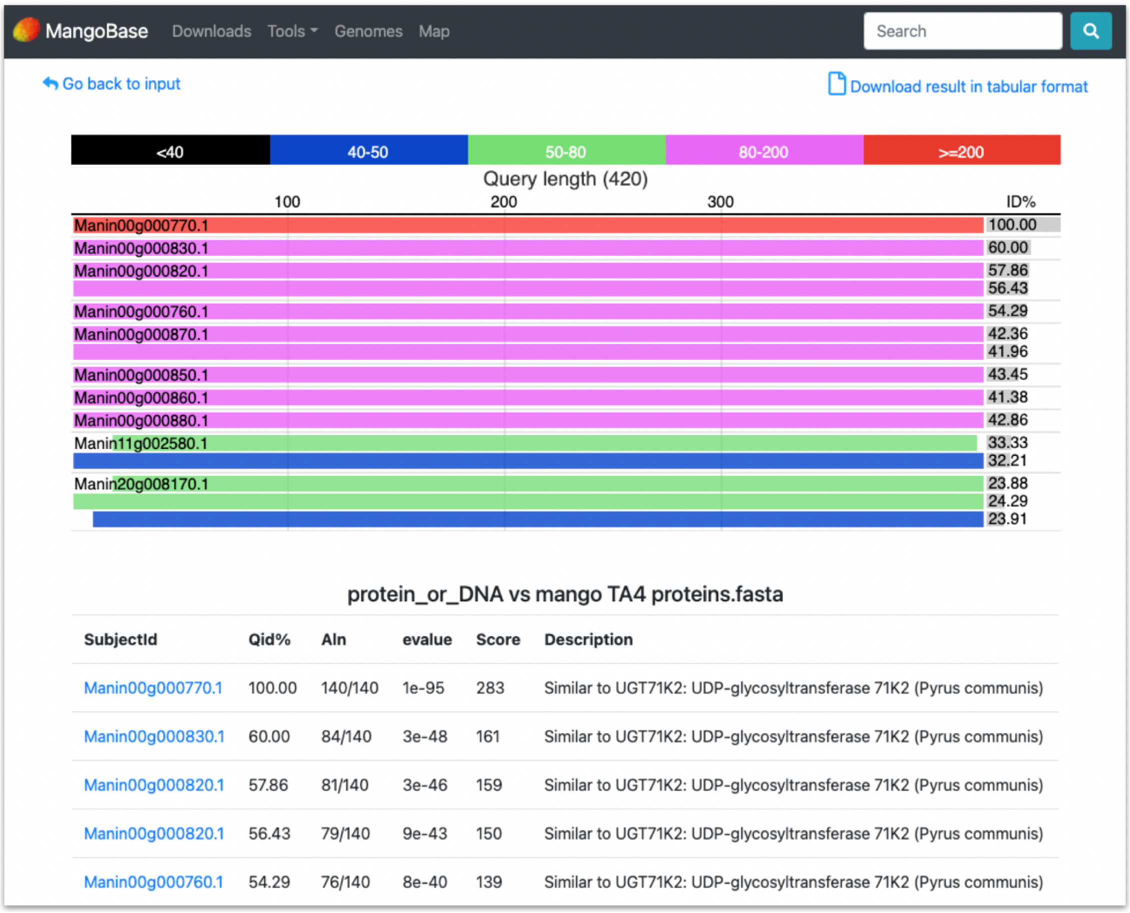

Figure S10. EasyGDB BLAST output page. Results can be downloaded in tabular format, genes are linked to the gene view (Figure S7), and alignments are available when scrolling down. A configuration JSON file can be used to define external links and links to genomic regions in the genome browser.

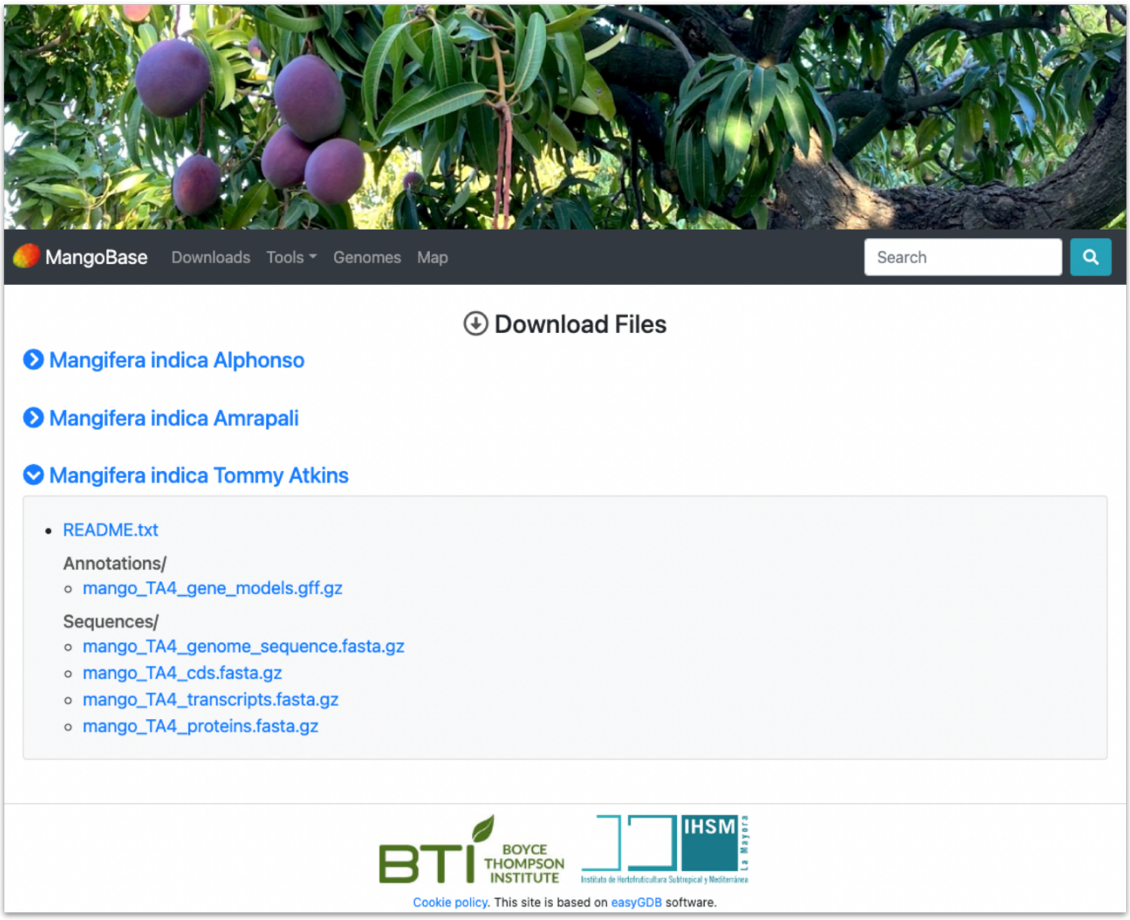

Figure S11. Example of the download section in MangoBase, which is implemented with EasyGDB. The organization of the links replicates the file organization of the downloads folder in EasyGDB.

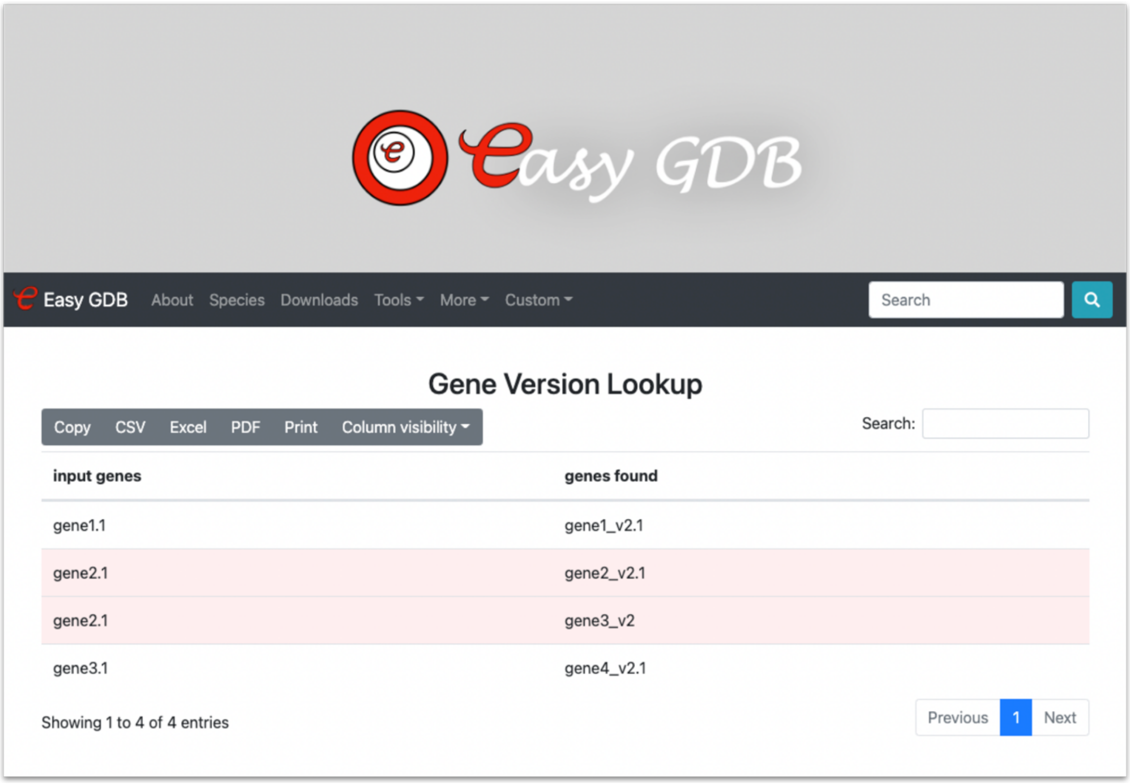

Figure S12. Example of the Gene version lookup tool. This tool allows users to query a list of identifiers to find their equivalent version in another list. It is useful to lookup gene ids between gene annotation versions or orthologs between related species. Identifiers with more than one equivalence are shown with a pink background.

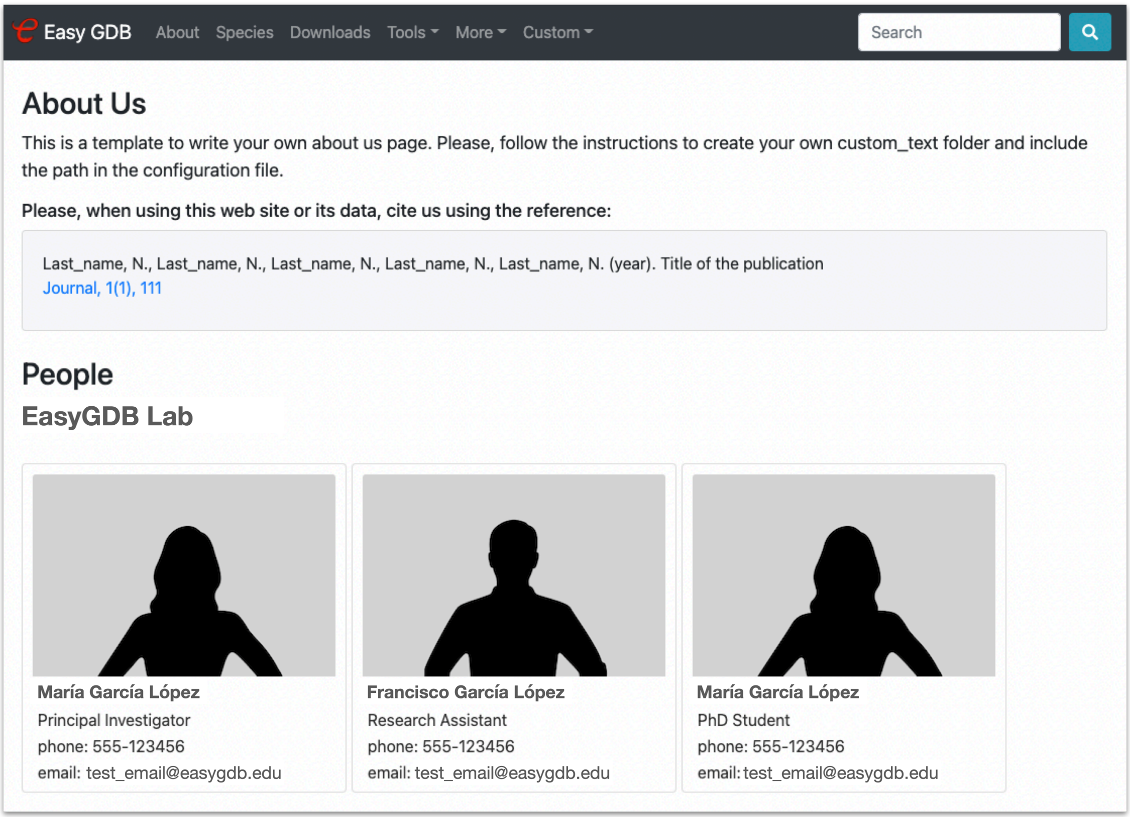

Figure S13. Example of About Us template in EasyGDB. It provides group cards which can be defined in a configuration JSON file.

References

Buchfink,B. *et al.* (2015) Fast and sensitive protein alignment using DIAMOND. *Nat. Methods*, **12**, 59–60.

Dunn,N.A. *et al.* (2019) Apollo: Democratizing genome annotation. *PLOS Comput. Biol.*, **15**, e1006790.

Humann,J.L. *et al.* (2019) Structural and Functional Annotation of Eukaryotic Genomes with GenSAS. In, Kollmar,M. (ed), *Gene Prediction*, Methods in Molecular Biology. Springer New York, New York, NY, pp. 29–51.

Jones,P. *et al.* (2014) InterProScan 5: genome-scale protein function classification. *Bioinforma. Oxf. Engl.*, **30**, 1236–1240.

Lyons,E. and Freeling,M. (2008) How to usefully compare homologous plant genes and chromosomes as DNA sequences: How to usefully compare plant genomes. *Plant J.*, **53**, 661–673.

Spoor,S. *et al.* (2019) Tripal v3: an ontology-based toolkit for construction of FAIR biological community databases. *Database*, **2019**, baz077.

Sterck,L. *et al.* (2012) ORCAE: online resource for community annotation of eukaryotes. *Nat. Methods*, **9**, 1041–1041.

Wilkerson,M.D. *et al.* (2006) yrGATE: a web-based gene-structure annotation tool for the identification and dissemination of eukaryotic genes. *Genome Biol.*, **7**, R58.
